# Statistical Learning Methods Applicable to Genome-Wide Association Studies on Unbalanced Case-Control Disease Data

**DOI:** 10.3390/genes12050736

**Published:** 2021-05-13

**Authors:** Xiaotian Dai, Guifang Fu, Shaofei Zhao, Yifei Zeng

**Affiliations:** Department of Mathematical Sciences, SUNY Binghamton University, Vestal, NY 13850, USA; xdai@binghamton.edu (X.D.); szhao@math.binghamton.edu (S.Z.); yzeng@math.binghamton.edu (Y.Z.)

**Keywords:** disease, GWAS, unbalanced case-control, genomic selection, genomic prediction

## Abstract

Despite the fact that imbalance between case and control groups is prevalent in genome-wide association studies (GWAS), it is often overlooked. This imbalance is getting more significant and urgent as the rapid growth of biobanks and electronic health records have enabled the collection of thousands of phenotypes from large cohorts, in particular for diseases with low prevalence. The unbalanced binary traits pose serious challenges to traditional statistical methods in terms of both genomic selection and disease prediction. For example, the well-established linear mixed models (LMM) yield inflated type I error rates in the presence of unbalanced case-control ratios. In this article, we review multiple statistical approaches that have been developed to overcome the inaccuracy caused by the unbalanced case-control ratio, with the advantages and limitations of each approach commented. In addition, we also explore the potential for applying several powerful and popular state-of-the-art machine-learning approaches, which have not been applied to the GWAS field yet. This review paves the way for better analysis and understanding of the unbalanced case-control disease data in GWAS.

## 1. Introduction

Over the past ten years, genome-wide association studies (GWAS) have shown great potential in investigating the biological and genetic etiology of disease, with the aims of providing better understanding, prevention, and treatment of diseases. As the cost of genotyping decreases, GWAS research moves to a new level, as phenome- wide association studies (PheWAS) enable thousands of phenotypes constructed from electronic health records (EHRs) and biobanks involving tens of millions of variants for hundreds of thousands of participants in large cohorts [1,2,3,4]. The PheWAS and large biobanks create new opportunities for detecting more scientific findings from the GWAS data [5,6]. However, most binary phenotypes have substantially fewer cases than controls [7].

Here we first give a few examples, from slight, moderate, to extreme imbalance ratios: The Wellcome Trust Case Control Consortium (WTCCC) provides a series of GWAS datasets that include 2000 case samples from each of seven common diseases [8] (e.g., type 1 diabetes [9], type 2 diabetes [10], coronary heart disease [10,11], bipolar disorder [12], rheumatoid arthritis [13,14], and so on). Their shared control has 3000 heathy samples (case-control ratio of 0.66). Dai et al. [15] analyzed a polycystic ovary syndrome (PCOS) affection status dataset consisting of 1043 cases and 3056 controls (with the case-control ratio of 0.34) and 731,442 SNPs.

The UK Biobank [1,16] is a very large study with over 400,000 participants from white British participants with European ancestry, which collected >1400 case-control disease phenotypes: colorectal cancer, prostate cancer, lung cancer, and Alzheimer’s, disease to name a few. Most of their binary phenotypes have a case-control ratio lower than 1:100 [1,7,16]. The Michigan Genomics Initiative (MGI) is taking efforts to create a biorepository of genomic data and has involved >25,000 samples with >500,000 SNPs. It has already been noticed that multiple phenotypes have an extremely small number of cases, as small as 20 cases [3,4].

The imbalance of binary phenotypes poses serious challenges to traditional statistical methods, in terms of both genomic selection [2,3,4,7,17,18,19,20,21,22,23,24,25,26] and phenotypic prediction such as the prediction on disease status [24,27,28]. In this article, we review several statistical methods that have been applied in the unbalanced case-control GWAS data. We commented on the advantages and disadvantages of each method; in addition, we also introduced some state-of-the-art machine-learning methods that have great potential to be applied to solve the imbalance issues in the GWAS field. These methods have received a lot of attention in many other fields but they have not been applied to analyze GWAS data yet. Sun et al. [29] also wrote an overview of statistical learning methods that are suitable for classification of unbalanced data; however, two major differences are: we mainly focused on the GWAS application that was out of the scope of Sun et al.; the statistical learning approaches introduced in this article represent the newer developments than those in [29].

This review article is organized as follows. We first discuss why the imbalance causes an issue from a statistical aspect. Then we introduce the generalized linear mixed model association test in Section 3, and the Scalable and Accurate Implementation of Generalized mixed model in Section 4. They are both single-SNP methods related to logistic mixed model. In Section 5, we comment the Bayesian multiple Logistic Regression method [24] as a joint variable selection method for binary traits. In the remaining sections, we comment on multiple state-of-the-art machine-learning algorithms, the Support Vector Machine [30] in Section 6, AdaBoost in Section 7, and neural network in Section 8. In Section 9, we introduce the permutation-based significance test skills to facilitate the usage of machine-learning approaches into the GWAS field. Finally, we discuss other challenges of GWAS data analysis and summarize the advantages and limitations of these approaches in Section 10.

## 2. Why Does the Imbalance Cause an Issue from a Statistic Aspect?

The problems caused by an imbalance in case-control data can be summarized from four aspects [31]: (1) erroneously assuming that the accuracy metric (e.g., error rate) is appropriate; (2) erroneously assuming that the distribution of test statistic is the same between the case and control group; (3) erroneously assuming that the minority group has an adequate sample size; (4) erroneously assuming that the underlying asymptotic assumptions are still valid.

Firstly, the error rate has been widely used as an accuracy metric in the classification literature. However, it averages all observations without treating them differently, under the assumption that the samples in minority class have equal importance as those in majority class. As a result, it always favors the majority class [32]. For example, if the data contain 99% of the control (negative) and 1% of the case (positive), then predicting everything as negative will give us 99% accuracy. Statistically speaking, the classifier works correctly if the accuracy metric were appropriate. Drummond et al. [33] showed that it is usually very hard to outperform this simple classifier if the data are unbalanced.

However, in the GWAS field with binary traits, people care more about the cases (the disease status) than controls (healthy). Therefore, it is more serious to misclassify a case compared to misclassifying a control. To overcome the problem of error rate, weighted loss function or AUC (the area under the ROC curve) has been used [34]. The ROC curve is a plot of true positive rate vs. false positive rate under various thresholds, and usually a higher AUC stands for a better classifier. Liang et al. [35] showed that AUC is statistically consistent and better than the error rate under many scenarios. Hanley et al. [36] showed that AUC is actually equivalent to the Mann–Whitney statistic. Since the AUC is more related with rank statistic, it is invariant to the prior probabilities, which makes it a desired accuracy metric in evaluating the unbalanced data.

Secondly, ideally speaking, the training distribution should be the same as the test distribution, but in the unbalanced case, it is relatively more likely to get different distributions between the training and test data when randomly splitting an unbalanced data set. For example, it is possible that the training data are highly unbalanced but that the test data are balanced; or in some extreme cases we may end up with no samples from the case group in training or test data. Under this circumstance, even when equipped with correct accuracy metric a classifier will not work well. To tackle this problem, sampling strategies such as over-sampling/under-sampling have been applied to make the data more balanced. However, over-sampling may increase the model and computation complexity, which is a burden for the GWAS data when millions of genetic variants are involved. Meanwhile, under-sampling may yield less information than we should have. See Zhou (2013) [37] for a detailed comparison on the performance of multiple sampling methods.

Thirdly, contamination may destroy the sample, and in particular for rare diseases it is always difficult to get enough case samples. When the number of minority classes is too small, we may have insufficient data for the classifier to learn, and thus yield bad results. As pointed out by Sammut and Webb [31], a ratio as low as 1:35 can make some methods inadequate for building a good model in some applications, while in some other situations, 1:10 may be tough to deal with. We should make different judgments based on different applications, datasets, sample sizes, and methods applied, etc.

Lastly, a very low case-control ratio in GWAS data may violate asymptotic assumptions of statistical inferences, such as that of the logistic regression models, which results in an inflated type I error rate [7]. For example, Chen et al. [2] assumes that the test statistic of genetic variants in a logistic mixed model asymptotically follows a Gaussian distribution under the null hypothesis, while the actual distributions are substantially different from Gaussian distribution when the case-control ratio is extremely unbalanced [7].

More specifically, unbalanced data may violate assumptions in statistical inferences. If the number of cases is drastically smaller than the number of controls, these cases may be viewed as outliers in most statistical models, and hence it leads to a higher variation for the estimation of coefficients. As a result, it shrinks the absolute value of test statistics and yields a larger *p*-value, which makes a truly influential variant insignificant.

Let us illustrate the idea using a simulation example. Suppose our sample size is 100 and we only have one true predictor *X*. In a balanced setting, we generate 50 controls from Uniform (0, 0.3) distribution, and remaining 50 cases from Uniform (0.7, 1). We then generate an intermediate variable *W* = *X* + ε where ε follows standard normal distribution. Finally, we connect the response with the only true predictor through an indicator function as

$$Y=I\left(W>0.6\right)=\left\{\begin{array}{c} 1,\;\mathrm{if}\;W\;>\;0.6,\\ 0,\;\mathrm{otherwise}. \end{array}\right.$$

In an unbalanced setting, we generate 90 controls from Uniform (0, 0.3), 10 cases from Uniform (0.7, 1), and follow the same procedure to generate the binary response Y. After the data are simulated, we use logistic regression to evaluate the significance of *X* in both balanced and unbalanced settings. We repeat 100 times and obtain the standard error and *p*-value of the coefficient of the true predictor *X*. From the results demonstrated in Table 1, we can see that even for this simple situation (with only one predictor), the *p*-value for the unbalanced data is almost ten times higher and it leads to a wrong conclusion that *X* is not significant.

## 3. Generalized Linear Mixed Model Association Test

Linear mixed models (LMMs) has become popular in GWAS for various biomedical traits because of its power in correcting for population structure and genetic relatedness [38,39,40,41,42,43,44,45]. Some fast algorithms have been proposed to estimate the model parameters and the variance component of LMMs to meet the ultrahigh dimensional need of the GWAS settings, such as the efficient mixed-model association (EMMA) [38], the efficient mixed models expedited (EMMAX) [39], and the fast linear mixed models (FaST-LMM) [41], to name a few. However, as a model with continuous response, LMMs are not designed for binary traits.

Chen et al. [2] applied logistic mixed models to analyze binary traits for GWAS data as follows

logit(*π_i_*) = *X_i_α* + *G_i_β* + *b_i_*,

where *π_i_* = *P*(*y_i_* = 1*|X_i_*, *G_i_*, *b_i_*); *y_i_* ∈ {1, 0} is the probability for a binary disease status phenotype to be a case for subject *i*, conditional on their covariates *X_i_*, genotype *G_i_*, and random effects *b_i_*. *X_i_* is a 1 *× p* row vector of covariates for subject *i*, *α* is a *p ×* 1 column vector of fixed covariate effects, *G_i_* is the genotype of a genetic variant for subject *i*, and *β* is the fixed genetic effect. The random effects *b* = {*b*_1_, *b*_2_,…, *b_n_*}*~N_n_*(0, *τV*), where *τ* is a scale variance component parameter and *V* is usually the genetic relationship matrix (GRM) estimated from a large number of genetic variants.

Chen et al. [2] also proposed a generalized linear mixed model association test (GMMAT) to select important genetic variants. The GMMAT score test is constructed based on the null hypothesis *H*_0_:*β* = 0, which leads to the same null logistic mixed model for all genetic variants: logit(*π_i_*) = *X_i_α* + *b_i_*. They fit the null logistic mixed model using the penalized quasi-likelihood (PQL) method [46] and the efficient AI- REML algorithm [47] to estimate the variance components. The algorithm will iteratively estimate the fixed effects *α* and random effects *b_i_* under the null hypothesis until the process reaches convergence. The score of each genetic variant under the null hypothesis is defined as $T=G\left(y-\hat{y}\right)$, where *G* = (*G*_1_, *G*_2_,..., *G_n_*)*^T^* is the *n* 1 column vector of genotypes, *y* = (*y*_1_, *y*_2_,..., *y_n_*)*^T^* is the *n* 1 column vector of observed outcomes, and $\hat{y}$ = ($\hat{y}$_1_, $\hat{y}$_2_,..., $\hat{y}$*_n_*)*^T^* is the estimated value of *y* under *H*_0_. The asymptotic *p*-value of each genetic variant is obtained by assuming that the test statistic *T* asymptotically follows a Gaussian distribution.

## 4. Scalable and Accurate Implementation of GEneralized Mixed Model

Zhou et al. [7] claimed that the type I error rate of GMMAT test can still be inflated under the presence of unbalanced binary traits because a very low case-control ratio may violate asymptotic assumptions of logistic regression models. They proposed a Scalable and Accurate Implementation of Generalized mixed model (SAIGE) based on the saddle point approximation (SPA) [48,49] to conduct the score test.

The SAIGE method still adopted the logistic mixed model structure from the GMMAT, but it improved the variance component and the test statistic to better account for imbalance of binary traits. Specifically, Zhou et al. [7] applied a state-of-the-art pre-conditioned conjugate gradient (PCG) approach [50,51] to solve linear systems for a large cohort without requiring the computation of GRM, which achieves faster iterations for large *n*. In addition, the SAIGE improved the calculation of the test statistic *T*. Specifically, the GMMAT test assumes that *T* asymptotically follows a Gaussian distribution under the null hypothesis, which only considers the first two moments (mean and variance). However, the underlying distribution of *T* can be substantially different from Gaussian distribution when the case-control ratio is unbalanced. The saddle point approximation is used to approximate the distribution of *T* using the entire cumulant generating function (CGF). Zhou et al. [7] claimed that the approximated CGF can provide a more accurate *p*-value than the GMMAT does under the presence of unbalanced binary phenotypes.

In simulation studies, Zhou et al. [7] showed that the GMMAT suffered from type I error inflation, but the SAIGE controlled the type I error rates effectively even when case-control ratios are extremely unbalanced (e.g., a case-control ratio of 1:99). Zhou et al. [7] also applied the SAIGE to the UK Biobank data, where most binary phenotypes have a case-control ratio around or lower than 1:100. For example, colorectal cancer has 4562 cases and 382,756 controls (with the case-control ratio of 0.012), glaucoma has 4462 cases and 397,761 controls (with the case-control ratio of 0.011), and thyroid cancer has 358 cases and 407,399 controls (with the case-control ratio of 0.0008). Although the analysis results of GMMAT suffered greatly from type I error inflation with no clear peaks in its Manhattan plot for the extremely unbalanced thyroid cancer phenotype (see Figure 1 of Zhou et al. [7]), the SAIGE approach successfully detected loci on Chromosome 9 that is well known for its association with thyroid cancer. Another data example with an extremely unbalanced case-control ratio is the MGI biorepository that is mentioned in the Introduction section. Dey et al. [3] analyzed four extremely unbalanced phenotypes from MGI by applying the SPA to approximate the *p*-value based on a test statistic that was also used in the SAIGE, and they reported a large number of significant markers whose nearby genes have been verified as truly associated with the corresponding phenotypes by other studies. Compared to SAIGE and GMMAT, the logistic regression model used by Dey et al. [3] did not exploit the random effect term.

Both the GMMAT and SAIGE are single-SNP methods. The single-SNP method assesses the potential association of each genetic variant in isolation from the others. As a result, multiple limitations have been found for single-SNP methods: they inflate both false-positive and false-negative results [52]; they have limited detection and prediction ability because most complex diseases are actually polygenetic [24,53,54,55,56], where multiple variants affect the disease simultaneously but each one may have weak individual association [24,57]; they fail to differentiate potentially causative from non-causative variants if there exists a strong linkage disequilibrium (LD) between the noise variants and the truly influential ones.

In the remaining sections, we review several joint methods that have the advantages of considering multiple SNPs simultaneously.

## 5. Bayesian Multiple Logistic Regression Method

Various Bayesian approaches have been applied to the GWAS field to make genomic selections [57,58,59,60,61,62,63]. The benefits of Bayesian approaches lie in that they consider the polygenic effects of a large-scale of SNPs in GWAS using only one joint model and provide a feasible solution to estimate a large amount of unknown parameters. For example, Zhou, Carbonetto, and Stephens [57] proposed the Bayesian Sparse Linear Mixed Model (BSLMM) to select truly influential genes from the high-dimensional GWAS data with *p >> n*, where sparsity or regularization is expected. The selection is processed by putting sparsity-enforcing priors on the regression coefficients so that the coefficients of non-influential genes can be forced to zero.

As an extension of BSLMM from the continuous to binary traits, a Bayesian multiple Logistic Regression method (B-LORE) was developed by setting point-normal priors on the regression coefficients:

*p*(*β_i_|φ_i_*, *σ*) = (1 *− φ_i_*)*δ*_0_ + *φ_i_ N*(*β_i_|*0, *σ*^2^),

where *δ*_0_ is a constant, the hyperparameter *φ_i_* controls the sparsity of the model, and *σ*^2^ is the variance of the regression coefficients of influential genes. For binary traits, the likelihood function for the logistic regression model is maximized as follows:

$$L\left(\beta \right)=\prod_{j=1}^{n}p_{j}{}^{y_{j}}{\left(1-p_{j}\right)}^{1-y_{j}},$$

where *y_j_* ∈ {1, 0} is the observed phenotype for the *j*th subject, and *p_j_* is the probability for a subject to have the disease given his/her genotypes and estimated regression coefficients *p_j_* = *p* (*y_j_* = 1 |*G*, $\hat{\beta }$). To estimate the hyperparameters *φ_i_* and *σ*, Banerjee et al. [24] calculated the marginal likelihood function instead of the full likelihood function so that ***β*** can be integrated out in their posterior conditional distribution. This approach can greatly reduce the number of parameters to be estimated and can eventually improve its computational efficiency.

Unlike the *p*-values in single-SNP models, Bayesian approaches use a binary latent vector, say ***c***, to assess the importance of each SNP, with *c_i_* = 1 meaning the *i*th SNP is influential and *c_i_* = 0 meaning the *i*th SNP is not influential. See Banerjee et al. [24] for details about how the latent vector is incorporated and estimated through the joint regression model and MCMC sampling. Furthermore, Banerjee et al. [24] demonstrated that the B-LORE method outperforms other Bayesian variable selection methods through simulated data with case-control ratios as low as 0.25. Banerjee et al. [24] applied the B-LORE approach to the German Myocardial Infarction Family Study data with 6234 cases and 6848 controls of white European ancestry, which has a relatively balanced case-control ratio. For extremely unbalanced GWAS data, the priors of the B-LORE may require further adjustments.

However, it is also a legitimate concern that a Bayesian regression is much more computationally inefficient than the single-SNP GWAS methods due to the large number of parameters and the long iterative sampling process. There are many ways to improve the efficiency of a Bayesian algorithm, among which the most standard approach is to use conjugate priors and fast Gibbs sampling applied on the unknown parameters [64]. Some others tried to reduce the computational cost of a Bayesian regression from multiple aspects, such as the fast estimation of covariance matrices [65] and the use of marginal likelihood of predictors instead of a full model likelihood [66,67], to name a few.

## 6. Support Vector Machine

The Support Vector Machine (SVM) [30] is a well-known machine-learning algorithm designed for classification of binary traits. It aims to locate an optimal hyperplane from the high-dimensional predictor space to separate the two classes of binary traits. The separation is achieved by maximizing the minimum of the distances of every data point to the hyperplane (defined as *r_j_*, *j* = 1,…, *n*). The result of SVM is interpretable because it tracks which genetic variants are used to construct the separating hyper-plane and classify binary traits. However, SVM may suffer from overfitting and loss of generalizability for high-dimensional data.

Marron et al. (2007) [68] proposed the Distance Weighted Discrimination (DWD) for classifications on high-dimensional and low sample-sized data (*p >> n*), which facilitates the applicability of SVM algorithm to case-control disease data in the GWAS fields. The DWD improves the standard SVM by locating the hyperplane that minimizes the sum of the reciprocals of *r_j_* (i.e., $\min\overset{n}{\underset{j=1}{\sum }}\frac{1}{r_{j}}$). The DWD approach overcomes the challenges of high-dimensional classification by allowing all data points to have influences on the separating hyperplane, rather than considering only the point that is closest to the separating hyperplane.

However, as claimed by Qiao et al. [69], the DWD does not perform well on unbalanced data if the proportions of the two classes are quite different from each other. Qiao et al. [69] proposed a weighted sum of the reciprocals of *r_j_* ($\min\overset{n}{\underset{j=1}{\sum }}\frac{w_{j}}{r_{j}}$) as an improved objective function to locate the separating hyperplane, where *w_j_* is the weight of the *j*th sample. They demonstrated through simulations and real data examples that the weighted DWD yields accurate and robust prediction results under nonstandard situations such as unbalanced binary traits. Additionally, they also proved Fisher consistency for the weighted DWD approach to provide statistical guarantee (see Qiao et al. [69] for more detailed theoretical results). Since a naive classifier easily favors the majority class, a common strategy that address the imbalance is to assign a higher misclassification cost to the underrepresented minority class of the binary trait. Qiao and Liu [70] developed an optimal weighting scheme using the Bayes decision rule with Mean Within Group Error (MWGE) criterion to determine the weight, *w_j_*, of each sample.

## 7. AdaBoost

Ensemble learning based on decision trees has been effective in achieving a balance between overfitting and under-fitting and also reducing variance of predictions through aggregating prediction results of multiple classifiers [71]. Another advantage of decision tree-based algorithms is that the hierarchical structures of decision trees naturally considers epistasis among variants without requiring an explicitly model structure [72]. For example, Bagging [73], Random forests [74], and AdaBoost [75,76] are some of the most well-known decision tree-based ensemble learning algorithms. In particular, the AdaBoost algorithm is known to be powerful for classifying unbalanced binary traits and capable of reducing bias of single classifiers [77]. It puts more weights on the subjects that are most often misclassified by the preceding decision trees. The AdaBoost algorithm is comprised of a series of “weak” decision trees, but the final model can still yield promising prediction performances as long as each tree can learn additional information from a subset of the subjects that are not achieving good results in preceding decision trees.

Despite the fact that the AdaBoost method already put more weight on misclassified subjects, it still treats subjects of the binary traits equally: weights of misclassified (correctly classified) subjects are increased (decreased) by the same percentage no matter they come from the majority class or from the minority class. In order to support the under-represented minority class and truly address the unbalanced case-control issue, Sun et al. [77] adjusted AdaBoost by assigning higher misclassification costs to the subjects coming from the minority class than those of the majority class. See Sun et al. [77] for details about the calculation of subject weights.

## 8. Neural Network

Frasca et al. [78] proposed a cost sensitive neural network (COSNet) method, which can handle unbalanced responses by utilizing a suitable Hopfield Network and learning parameter through a cost sensitive optimization procedure. They also introduced a regularized cost sensitive neural network (RCOSNet) by adding a regularization term into the energy function of the network that can deal with extremely unbalanced classification problems. These two characteristics make RCOSNet applicable to case-control disease GWAS datasets. Zhang et al. [79] proposed a stacked de-noising Auto-encoder neural network (SDAE) algorithm based on cost-sensitive oversampling. Cost- sensitive oversampling exploited misclassification cost as the weight of the original data and duplicate samples based on that weight to maintain balance between different classes [80]. They added noises into the input features using a Gaussian distribution, or Salt and pepper distribution, and hence improved the classification accuracy of the minority class compared to traditional stacked Auto-encoder neural network.

**Figure 1:**
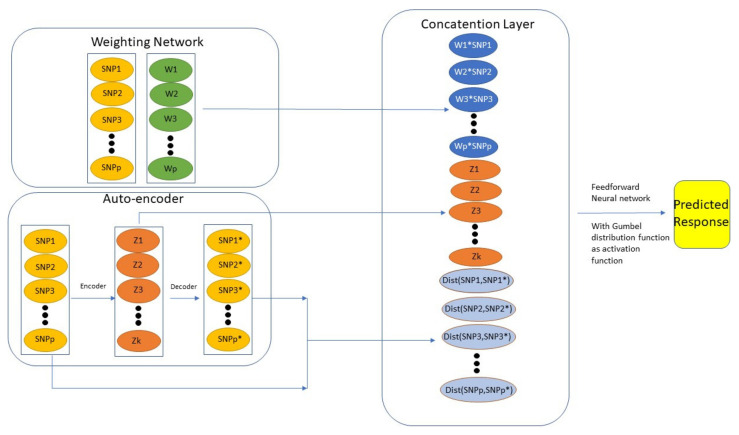
Overview of GEV-NN structure.

Munkhdalai et al. [81] proposed the Generalized Extreme Value distribution Neural Network (GEV-NN) that consists of three components: “Weighting Layer”, “Auto-encoder Layer”, and “Concatenation Layer”. The “Weighting Layer” gives weight to each predictor by multiplying a factor in front to each predictor. The “Auto-encoder Layer” extracts important features out from samples. The “Concatenation Layer” combined the previous two components and feed the result to the final prediction function. In order to overcome the imbalance issue, they used a Gumbel distribution as an activation function in the network. Figure 1 illustrates the model structure proposed by Munkhdalai et al. [81]. By using the Auto-encoder, they generate efficient features (which in this case is the distance between original inputs and reconstructed inputs) for minority classes (as shown in the “Concatenation Layer” of Figure 1). See Munkhdalai et al. [81] for more technical details. Actually, Kweon et al. [82] has already compared the GEV-NN to some baseline methods such as logistic regression, random forest, AdaBoost, XGBoost, and Support Vector Machine using a health-related dataset to predict hypertension. GEV-NN achieved the best prediction performance in terms of a number of evaluation metrics including G-mean, AUC, Accuracy, Brier score, and F score.

Another well-known variable selection mechanism for the neural network was the so-called “dropout” proposed by Srivastava et al. [83], which is one of the most highly cited machine-learning research methods. In order to avoid overfitting, Srivastava et al. [83] proposed to randomly drop features (including original predictors and engineered features) from the neural network during training process and evaluate their impacts on predictions to select an optimal “thinned” network. The “dropout” strategy can be regarded as regularization of neural networks including GEV-NN and can be used for variable selection purposes. Therefore, the “dropout” strategy has great potential for selecting influential variants from high-dimensional GWAS data. 

## 9. Significance Test

Performing the hypothesis test and statistical significance for each variant has been the core of the GWAS field, and, therefore, whether the *p*-value can be obtained is crucial to select significant genetic variants. However, the statistical significance study for the state-of-the-art machine-learning approaches is still under-developed. In order to facilitate wide applications of machine-learning approaches into the GWAS field, we refer the readers to the well-established permutation-based significance test skills, which have already been applied to some classifiers to obtain *p*-values [84,85]. For example, Chen et al. [86] performed the statistical significance test by permuting variable importance scores obtained from the random forests approach to obtain *p*-values [74]. As a non-parametric approach, the permutation-based statistical significance test can be applied to the SVM (Section 6), AdaBoost (Section 7), and Neural Network (Section 8) to obtain a *p*-value for each variant.

The distribution of a test statistic is empirically established by permuting the original data with a large amount of time. Then an empirical *p*-value of each variant is approximated by counting the fraction of the test statistic scores of permuted data that are larger than that of the original data [84,85]. The accuracy of *p*-values depends on the original data (whether there exist any real associations between variants and binary response) as well as the classifier itself (whether the classifier is able to discover these associations) [84]. Actually, prediction and significance studies are related. Specifically, a promising prediction performance of a machine-learning method can be an indicator of a good understanding of the dependency structure between the predictors and the response, which is very important for constructing a reliable and powerful significance test [84]. See Ojala and Garriga [84] for more details about the theoretical properties of permutation-based tests.

## 10. Conclusions

With the collection of a large-scale of diseases from participants in large cohorts such as biobanks and EHRs, it raises rapidly increasing demands on statistical and machine-learning methods driven by unbalanced case-control GWAS data analysis needs. In this article, we reviewed multiple methods that are designed to address the imbalance in binary traits, including GMMAT and SAIGE that were based on logistic mixed models, the Bayesian variable selection method B-LORE, and machine-learning approaches such as SVM, AdaBoost, and the neural network. Each method has its own advantages as well as limitations, as summarized in Table 2. It is impossible to find any method that is uniformly the best. Therefore, methods should be chosen according to the needs of different aims, backgrounds, and scientific questions for different datasets. For example, if one wants to build a high-performance disease risk prediction tool along with a ranking of the most influential genetic variants meanwhile taking care of the nonlinear and gene-gene interactions, the AdaBoost algorithm along with its variable importance measure will be an ideal option.

In addition to the unbalanced classification issue, there are several other common challenges and concerns in the GWAS literature, to name a few in the following: (1) The GWAS data easily involves millions of SNPs for only thousands of participants, the ultrahigh-dimensionality, “small *n* big *p*” or the curse of dimensionality issue, raise big challenges [87]. (2) LD is one of the most important, extensive, and widespread features in genomes, with 70–80% of genomes showing regions of high LD. As a result, it is difficult to separate the individual variants that are truly causative from those confounding spurious variants that are irrelevant to the phenotype but highly correlated with the causative loci due to LD [52]. (3) Epistasis is defined as nonlinear interactions among loci or among genes (GxG), has been gaining more and more attention for its substantial role in regulating biological traits [88,89,90,91,92,93,94,95,96]. (4) The underlying population structure acting as confounders in GWAS data [72]. (5) A method that is computationally efficient is desired in the GWAS field due to the extremely high volume of computational needs. 

To overcome the ultrahigh dimensionality challenge, Carlsen et al. [52] proposed a two-stage framework to extensively eliminate a large amount of noise SNPs using feature screening skills (its theoretical sure screening consistency is guaranteed), and then applied a sophisticated model to analyze the remaining variables in depth. They demonstrated that the accuracy and speed of genomic selection from the whole-genome data using this two-stage approach outperformed the approaches that applied only logistic ridge regression model or only a single-SNP approach. This two-stage framework is flexible enough to bridge any machine-learning approaches introduced in this article with the sure independence screening (SIS) feature screening approaches so that the performance of machine-learning approaches is not affected much by the ultrahigh dimensionality.

In addition to genomic selection, phenotypic prediction such as prediction of disease status or population disease prevalence using GWAS data repositories have also attracted a lot of research attention lately [24,27,28,97,98]. For example, Banerjee et al. [24] tried to predict the risk of coronary artery disease (CAD) for participants with white European ancestry. We want to emphasize that prediction has been the focus and strength of machine-learning approaches. However, significance- and inference-related research is still under-developed in machine-learning fields. We hope that this article highlights the importance of incorporating machine-learning approaches into the GWAS field, so that significance- and inference-related research could be improved in machine-learning approaches in the future.

## Figures and Tables

**Table 1:** The mean (standard error) of the simulation example across 100 replications.

Simulation Settings	Standard Error	*p*-Value
Balanced data	0.5956 (0.0275)	0.0275 (0.0689)
Unbalanced data	0.9731 (0.1410)	0.1916 (0.2664)

**Table 2:** A summarization of the methods evaluated from different aspects mentioned in the manuscript. Each method has its own advantages and limitations.

	Can the Method Be Applied to Genomic Selections?	Can the Method Be Applied to Genomic Predictions?	Can the Method Handle Unbalanced Binary Response?
GMMAT	√ GMMAT is designed for performing the significance test of each variant.	✘ GMMAT is a single-SNP method and is not good for prediction.	✘ Its significance test assumes a Gaussian distribution, which is not the case for unbalanced data.
SAIGE	√ SAIGE is designed for performing the significance test of each variant.	✘ SAIGE is a single-SNP method and is not good for prediction.	√ SAIGE use the entire cumulant generating function to approximate *p*- values.
B-LORE	√ B-LORE is a joint Bayesian variable selection regression method designed for high-dimensional variants.	√ B-LORE is a joint Bayesian regression and can be used for prediction.	✘ B-LORE cannot handle extremely unbalanced binary data.
SVM	√ SVM has not been widely used in GWAS field yet, but it has the potential to select important variants or use permutation-based testing to obtain significance.	√ SVM is a machine method with the strength of producing accurate prediction.	√ SVM with weighted DWD can handle extremely unbalanced binary data.
AdaBoost	√ AdaBoost has not been widely used in GWAS field yet, but it has the potential to select important variants or use permutation-based testing to obtain significance.	√ AdaBoost is a machine method with the strength of producing accurate prediction.	√ AdaBoost can handle extremely unbalanced binary data by assigning higher misclassification costs to the minority class.
Neural Network	√ Neural Network has not been widely used in GWAS field yet, but it has the potential to select important variants or use permutation-based testing to obtain significance.	√ Neural Network is a machine method with the strength of producing accurate prediction.	√ The RCOSNet and GEV-NN can handle extremely unbalanced binary data.

## Data Availability

Not applicable.
